# Genotype-specific molecular and functional trajectories in NGLY1 deficiency: a comparative multi-omics study of patient-derived iPSC neurons

**DOI:** 10.3389/fcell.2026.1966226

**Published:** 2026-10-15

**Authors:** Soukaina Amniouel, Zeenat A. Shyr, William F. Borschel, Mitali Tambe, Atena Farkhondeh, Wei Zheng

**Affiliations:** National Center for Advancing Translational Sciences, National Institutes of Health, Bethesda, MD, United States

**Keywords:** genotype-specific biomarkers, rare diseases, induced pluripotent stem cells, multielectrode array, NGLY1 deficiency, proteomics, transcriptomics, multi-omics

## Abstract

**Background:**

NGLY1 deficiency is an ultra-rare congenital disorder of deglycosylation with prominent neurodevelopmental impairment and marked allelic heterogeneity. Prior human neuronal models have characterized NGLY1 loss largely within single genetic backgrounds, leaving open whether distinct patient genotypes converge on a shared molecular state or follow divergent routes. Because clinical presentations overlap across genotypes, resolving this question requires a direct, within-system comparison of multiple genotypes rather than characterization of any single line.

**Methods:**

We generated iPSC-derived glutamatergic neurons from three patients carrying distinct biallelic NGLY1 genotypes (R401X/R401X, Q208X/G310G, L318P/R390P) and two healthy controls, and profiled them head-to-head using matched transcriptomics and proteomics at day 28, RNA to protein concordance and differential exon usage analysis, pathway and phenotype network integration, and longitudinal multielectrode array (MEA) recordings during early network maturation.

**Results:**

Across both molecular layers, samples segregated strongly by genotype rather than forming a unified disease signature. Differential expression revealed pronounced between-genotype heterogeneity with only a small shared molecular core. RNA to protein concordance was moderate and genotype-dependent, and differential exon usage was enriched among discordant genes in a mutation-dependent manner, implicating splicing-level regulation in the transcript to protein divergence. Integrated networks linked genotype-specific concordant changes to synaptic signaling, calcium handling, metabolism, and extracellular matrix remodeling, and connected them to overlapping clinical phenotypes through distinct molecular routes. MEA recordings showed delayed maturation and genotype-dependent shifts in network excitability, with functional clustering that mirrored the molecular separation.

**Conclusion:**

Distinct NGLY1 genotypes produce divergent molecular and functional trajectories that converge on impaired network maturation through non-identical routes. These findings reframe NGLY1 deficiency as a set of genotype-specific molecular states rather than a single phenotype, with direct implications for mechanistic subgrouping and the design of preclinical models and validation studies.

## Background

NGLY1 deficiency, or NGLY1-related congenital disorder of deglycosylation, is an ultra-rare autosomal recessive disorder caused by biallelic loss-of-function variants in NGLY1, which encodes the cytosolic deglycosylase N-glycanase 1 (Dabaj et al., 2021; Pandey et al., 2022). NGLY1 participates in endoplasmic reticulum-associated degradation (ERAD) by removing N-linked glycans from misfolded glycoproteins following retrotranslocation to the cytosol (Pandey et al., 2022; Enns et al., 2014). Clinically, the disorder presents as a multisystem neurodevelopmental syndrome featuring developmental delay, movement abnormalities, alacrima, seizures, peripheral neuropathy, and variable liver involvement (Enns et al., 2014; Zhang et al., 2025; Tong et al., 2023). Neurological impairment is prominent, whereas hepatic manifestations are variable and often transient (Zhang et al., 2025; Tong et al., 2023).

At the molecular level, NGLY1 loss disrupts proteostasis and impairs activation of NFE2L1, a transcription factor required for proteasome recovery after proteotoxic stress (Pandey et al., 2022; Tomlin et al., 2017). Defective NGLY1-NFE2L1 signaling has been linked to impaired proteasome gene induction, mitochondrial dysfunction, altered mitophagy, and inflammatory signaling (Forcina et al., 2022). These processes are particularly relevant to neurons, which depend on precise protein turnover, organelle quality control, and synaptic homeostasis (Tomlin et al., 2017; Tanaka and Matsuda, 2014).

NGLY1 deficiency also displays marked allelic and clinical heterogeneity, and genotype-phenotype relationships remain incompletely resolved in patient cohorts. The allelic spectrum includes nonsense, missense, frameshift, and splice-altering variants, with recurrent alleles such as p. R401X reported across multiple patients. This heterogeneity raises the possibility that similar clinical presentations arise from distinct underlying molecular states (Stanclift et al., 2022).

Human neuronal models have begun to define the consequences of NGLY1 loss, but these characterizations have largely been performed within single genetic backgrounds. Patient-derived neuronal stem cells carrying the R401X mutation, compared against CRISPR-corrected isogenic controls, show elevated ER stress, oxidative stress, and autophagic flux (Shyr et al., 2025), whereas motor neuron and neuromuscular junction models exhibit altered electrophysiology and impaired synaptic function (Manole et al., 2023). Importantly, Manole et al. used patient-derived cortical neurons within functional-gene rescue to isolate NGLY1-attributable disruptions in protein aggregate handling, mitochondrial homeostasis, and synaptic function (Manole et al., 2023). Such rescue-based designs are well suited to identifying effects attributable to NGLY1 within a fixed background, but they leave a distinct question unresolved: given the marked allelic heterogeneity of NGLY1 deficiency and the overlapping clinical presentations across patients, do distinct genotypes converge on a shared neuronal molecular state, or do they follow divergent routes to a common clinical spectrum? Answering this requires a direct, within-system comparison of multiple genotypes rather than characterization of any single line.

Here, we address this gap by comparing iPSC-derived glutamatergic neurons from three patients carrying distinct biallelic NGLY1 variants (R401X/R401X, Q208X/G310G, and L318P/R390P) alongside healthy controls, profiled head-to-head using matched transcriptomics and proteomics at day 28, RNA-protein concordance analysis, integrated pathway-phenotype network mapping, and longitudinal MEA recordings during early network maturation. Rather than seeking to isolate NGLY1-attributable effects within one background, we ask whether distinct genotypes produce shared or divergent molecular states and whether those states map onto-line specific network phenotypes. To our knowledge, this is the first head-to-head comparison of multiple NGLY1 genotypes within a single neuronal system, and it defines the genotype-specific molecular architecture underlying impaired neuronal maturation in NGLY1 deficiency.

## Materials and methods

### Patient-derived iPSC lines, glutamatergic neurons, and NGLY1 mutation characterization

Patient iPSC lines derived from three individuals harboring distinct biallelic mutations in *NGLY1* were chosen for this study. All lines were validated for pluripotency and genomic integrity prior to differentiation (Pradhan et al., 2021; Yang et al., 2019; Li et al., 2019). Two independent healthy control iPSC lines (WC31 and BD3) were used as references. The primary control (WC31) was used across all analyses, including immunostaining, transcriptomics, proteomics, differential exon usage, and multielectrode array recordings. The second control (BD3) was included specifically to strengthen the statistical comparisons in transcriptomics and proteomics analyses and was therefore incorporated into the omics differential expression contrasts only. Human iPSC-derived cortical glutamatergic neurons were obtained as differentiated preparations from BrainXell (https://brainxell.com) and cultured under the conditions described below to support functional maturation. The neuronal cultures were not subjected to additional post-differentiation sorting or purification in our laboratory and are therefore described as enriched for glutamatergic neurons rather than as a fully purified glutamatergic neuronal population. The three disease-causing NGLY1 variants represent a continuum of functional disruption (Table 1). Three independent biological replicates were used per genotype and per control line.

**TABLE 1:** Genotypes and predicted functional impact of NGLY1 mutations in patient-derived iPSC lines, and healthy control iPSC lines used as references.

Line id	Genotype (DNA)	Protein change	Mutation type	Domain affected	Predicted mechanism
594D	953T>C/1169G>C	L318P/R390P	Missense/Missense	TG domain	Destabilized protein or reduced activity
519D	622C>T/930C>T	Q208X/G310G	Nonsense + splice-affecting	Pre-TG/splice junction	Partial loss – truncated or aberrant splicing
592C	1201A>T/1201A>T	R401X/R401X	Nonsense (homozygous)	PNGase domain	Null – no functional protein
Control 1 (WC31)	Wild-type	N/A	Healthy control	N/A	N/A
Control 2 (BD3)	Wild-type	N/A	Healthy control	N/A	N/A

### Neuronal culture, maintenance and immunostaining

Upon thawing, 50,000 viable neurons were seeded into poly-D-lysine (PDL)-coated 96-well plates. Neurons were cultured for 28 days to allow functional maturation and maintained according to BrainXell’s recommendations, with media replaced twice weekly for 28 days. Neurons were fixed with 4% PFA for 15 min at room temperature, followed by permeabilization with 0.1% Triton X-100 for 10 min. After blocking in cell staining buffer (BioLegend) for 1 hour, wells were incubated overnight at 4 °C with primary antibodies–against MAP2 (1:1,000 dilution; Abcam #ab5392) and vGLUT2 (1:200 dilution; ThermoFisher #MA5-27613). Wells were washed 3 times with PBS, then incubated for 1 hour with goat anti-chicken Alexa Fluor 555 and goat anti-mouse Alexa Fluor 488 secondary antibodies. After three additional PBS washes, cells were counterstained with 4 µM Hoechst 33342 (Thermo Fisher Scientific) in DPBS for 30 min at room temperature. Cells were washed with PBS twice before sealing the plates and imaging on an Opera Phenix Automated imager (Revvity) using ×20 or ×63 water objective.

### RNA-seq data processing and differential expression analysis

Total RNA was isolated from patient-derived iPSC glutamatergic neurons and subjected to RNA sequencing. Library preparation, sequencing, and primary bioinformatic processing were performed by GENEWIZ/Azenta. Samples were sequenced on an Illumina platform in a 2 × 150 bp paired-end configuration. Sequence reads were quality- and adapter-trimmed with Trimmomatic v.0.36, aligned to the *Homo sapiens* GRCh38 reference genome (Ensembl) with the splice-aware aligner STAR v.2.5.2b to produce genomic BAM alignments, and summarized to gene-level counts with featureCounts from the Subread package v.1.5.2 using the Ensembl gene_id annotation and counting only uniquely mapped reads within exonic regions. This alignment-plus-summarization workflow was used rather than a transcript-level pseudoalignment approach, and all differential expression testing was therefore performed on the gene-level integer count matrix.

Differential expression analysis was performed in R (v.4.6.0) using edgeR. Ensembl identifiers were reduced to their base form, and counts for any duplicated identifiers were summed. Genes with low expression were removed with filterByExpr using a minimum count of 10, and library sizes were normalized by the trimmed mean of M values (TMM) method. A single quasi-likelihood generalized linear model was fitted across all libraries with one coefficient per cell line (design ∼0 + cell line), with dispersions estimated using the robust option and the model fitted with glmQLFit. Each NGLY1 patient genotype was compared with the equally weighted mean of the two healthy control lines (Control 1, WC31; Control 2, BD3) using a defined contrast in which each disease line received a weight of +1 and each control line a weight of −1/2, so that both controls contributed equally to every comparison. Significance was assessed with the quasi-likelihood F test (glmQLFTest). Genes with a Benjamini–Hochberg adjusted p value (FDR) below 0.05 and an absolute log_2_ fold change of at least 1 were considered differentially expressed.

### Proteomics data processing and differential expression analysis

Global protein profiling was performed by Poochon Scientific (Rockville, MD) using tandem mass tag (TMT) multiplex labeling and liquid chromatography with tandem mass spectrometry (LC-MS/MS). Protein lysates were prepared from each sample, and protein concentration was determined using a BCA reducing-reagent-compatible assay kit (Thermo Scientific). For each sample, 100 µg of protein was reduced with TCEP, alkylated with iodoacetamide for 30 min at room temperature in the dark, and digested with trypsin at 37 °C overnight (16 h). Tryptic digests were labeled using TMT18plex reagents according to the manufacturer’s instructions. Labeled peptide mixtures were fractionated into 24 fractions by basic reverse-phase liquid chromatography on an Agilent 1,290 Infinity UHPLC system fitted with a Poroshell HPH-C18 column (2.1 × 150 mm, 2.7 µm; Agilent), and each fraction was analyzed by a 110-min LC-MS/MS run.

Nanospray LC-MS/MS analysis was performed on a Thermo Scientific Exploris 480 with a Vanquish Neo RSLCnano system, or an Exploris 240 Orbitrap with an Ultimate 3000 RSLCnano system. Peptides were loaded onto a trap cartridge and eluted onto a 25 cm C18 EasySpray column using a linear acetonitrile gradient (3%–36%) in 0.1% formic acid over 110 min at 0.3 μL/min, then ionized using a Nano EasySpray ion source (spray voltage 1.8 kV, capillary temperature 275 °C). The instrument was operated in data-dependent mode. Full-scan MS spectra (m/z 350–1800) were acquired in the Orbitrap at 60,000 resolution (m/z 200) with an AGC target of 3 × 10^6^ and a maximum injection time of 100 ms. The 20 most intense multiply charged ions (z ≥ 2) were sequentially isolated and fragmented by higher-energy collisional dissociation (normalized collision energy 30, AGC target 1 × 10^5^, maximum injection time 400 ms, resolution 17,500, isolation window 1.2). Dynamic exclusion was set to 20 s, and charge-state screening rejected unassigned, 1+, and ≥7+ ions.

Raw data were searched against the UniProtKB protein sequence database using Proteome Discoverer (Version 2.5, Thermo Fisher) with the SEQUEST and Percolator algorithms, applying a 1% false discovery rate. Relative protein abundance was calculated as the ratio of sample abundance to reference abundance from summed reporter-ion intensities, normalized to one TMT tag or the master-mix tag.

Downstream differential-abundance analysis was performed in R (v.4.6.0). Normalized protein abundances from the reference-normalized reporter-ion block were log_2_ transformed and centered on the per-sample median, and no imputation was applied. Proteins were retained if they were assigned to *H. sapiens*, had a protein identification q value of 0.01 or less, were supported by at least two peptides, and were completely quantified across all analyzed samples; entries lacking a valid gene symbol or beginning with an Ensembl gene identifier were excluded. A single representative protein group was retained per gene symbol, selected by peptide count, unique-peptide count, and identification q value independently of differential significance. Differential abundance was assessed with limma using a linear model with one coefficient per cell line (design ∼0 + line) fitted across all samples, followed by contrasts. fit and empirical Bayes moderation with the trend and robust options enabled. Each NGLY1 patient genotype was compared with the equally weighted mean of the two healthy control lines (WC31 and BD3) using a defined contrast. Proteins with an FDR below 0.05 and an absolute log_2_ fold change of at least log_2_(1.5) = 0.585 were considered differentially abundant.

### Integrated transcriptome and proteome analysis of RNA to protein attenuation

Mutation-specific edgeR RNA-seq and limma proteomics results were integrated for 519D, 592C, and 594D, each compared with the equally weighted mean of the two controls, without refitting the primary models. Features were matched by gene symbol after removal of invalid or Ensembl-like symbols. When more than one RNA gene or protein group mapped to the same symbol, one representative was selected independently of differential significance, by highest logCPM for RNA-seq and by peptide count, unique-peptide count, identification q value, and abundance for proteomics. The gene products quantified in both assays defined the matched cross-omics universe.

Primary RNA DEGs were defined as FDR below 0.05 and absolute log_2_ fold change of at least 1, whereas differentially abundant proteins were defined as FDR below 0.05 and absolute log_2_ fold change of at least log_2_(1.5). For direct assay comparison, both datasets were additionally evaluated under the harmonized threshold of FDR below 0.05 and absolute log_2_ fold change of at least 0.585, and sensitivity was assessed across a range of FDR and effect-size cutoffs. Proteomic coverage was evaluated across transcript-expression deciles, and each matched gene product was assigned to a regulation class capturing concordant significance, discordant significance, protein-only, RNA-only attenuated, RNA-only equivalent or stable, or neither. Equivalence was defined as a 90% protein confidence interval falling entirely within ± 0.585 log_2_ fold change. RNA to protein concordance was summarized using Spearman and Pearson correlation, direction agreement among features significant in both assays, and the Jaccard overlap of the significant sets, computed on uncapped effect sizes. Deming regression with an equal error ratio, Deming slope uncertainty from 5,000 paired bootstrap resamples, and expected differentially abundant protein counts under RNA-sized effects from 500 simulations. Analyses were performed in R 4.6.0 under a fixed random seed.

### Differential exon usage analysis

Differential exon usage was assessed independently for 519D, 592C, and 594D relative to control WC31 using DEXSeq. For each comparison, exon-counting-bin counts from the three disease replicates and the three WC31 control replicates were analyzed after confirming that the exon-bin annotation and the control counts were identical across the three input files. Unlike the transcriptomic, proteomic, and integrative analyses, which used both healthy control lines, the differential exon usage analysis used Control 1 (WC31) alone, because exon-bin-level count data required for DEXSeq were not available for Control 2 (BD3).

Counting bins were retained when they carried at least 10 reads in at least three of the six samples in a comparison, and a gene or aggregate group was required to retain at least two such bins; the DEXSeqDataSet was constructed before filtering so that the gene-level total for each tested bin reflected all original bins of the gene. Size factors and dispersions were estimated with DEXSeq, and differential exon usage was tested by comparing the full model ∼ sample + exon + condition:exon with the reduced model ∼ sample + exon. Bin-level p values were adjusted by the Benjamini–Hochberg procedure and gene-level q values were computed with perGeneQValue, with significance defined at FDR below 0.05 at both the bin and gene level. Aggregate groups spanning more than one Ensembl gene identifier were retained but flagged as ambiguous and excluded from overlap analyses. Relative exon-bin usage fold changes were estimated with estimateExonFoldChanges. Enrichment of differential exon usage among RNA to protein discordant genes was tested with Fisher exact tests against all other matched, DEXSeq-tested genes, with Benjamini–Hochberg correction across comparisons. NGLY1 and XBP1 were examined as prespecified genes; because the IRE1-mediated XBP1 event is a 26-nucleotide unconventional intron below the resolution of exon-bin counting, the XBP1 exon-bin result is reported as suggestive rather than as a direct measure of the spliced-to-unspliced ratio.

### Cross-omics pathway concordance

Pathway over-representation analysis was performed within the matched universe of jointly tested gene products so that RNA and protein enrichment were assessed over an identical background. For each genotype, differentially expressed genes, differentially abundant proteins, and concordant markers (features significant in both assays in the same direction) were partitioned into marker sets comprising all significant features and, separately, the upregulated and downregulated subsets, and each set was tested independently. Gene symbols were mapped to Entrez identifiers with org. Hs.e.g.,.db, and enrichment was computed with clusterProfiler using compareCluster, calling enrichGO for the Gene Ontology Biological Process, Molecular Function, and Cellular Component ontologies, and ReactomePA enrichPathway for Reactome, each against the matched Entrez background and restricted to gene sets of 10–500 genes. Benjamini–Hochberg correction was applied within each marker set and pathway database, and enrichment was considered significant at FDR below 0.05. Redundant Gene Ontology terms were reduced with clusterProfiler simplify at a semantic similarity cutoff of 0.70. For display, pathways were prioritized by the number of layers in which they reached significance and then by FDR, terms were mapped to curated biological programs, and the signed −log10 FDR (positive for upregulated and negative for downregulated marker sets) was shown with circle size encoding the number of overlapping markers.

### Mutation–pathway–symptom network integration

To relate the shared and genotype-specific molecular states to the clinical features of NGLY1 deficiency, an integrative network was constructed linking mutation line, molecular marker, enriched pathway, and NGLY1 symptom module. Markers were classified within each molecular layer (RNA-seq, proteomics, and concordant RNA and protein) as common to all three lines, pairwise shared, or mutation specific. Markers were linked to enriched pathways in which they contributed to a significant over-representation result, and to NGLY1 symptom modules derived from Human Phenotype Ontology disease annotations (OMIM:615273 and ORPHA:404454), using the current phenotype. hpoa and phenotype_to_genes releases consolidated into curated high-level clinical modules. Marker-to-symptom edges are ontology-based gene-phenotype annotations rather than associations measured in these neurons, and are used only for hypothesis generation without causal or diagnostic inference. For the symptom-module analysis specifically, a more permissive protein significance threshold (FDR below 0.1 and absolute log_2_ fold change of at least log_2_(1.25)) was applied, because the stricter proteomic cutoff yields concordant marker lists too small for reliable over-representation testing. Markers were prioritized by a composite score combining cross-line molecular evidence with connectivity to the pathway and symptom layers; this is a ranking heuristic and not a significance value. Networks were assembled and summarized with igraph and exported as complete node and edge tables and GraphML files, with displayed figures showing an evidence-ranked subset for legibility. Because each mutation is represented by a single patient-derived line, mutation-specific findings may include cell-line background effects.

### Multielectrode array (MEA) culture and recordings

MEA cultures and recordings were conducted by BrainXell Therapeutics (https://brainxell.com) in accordance with their internal protocols. 60,000 viable neurons of each cell line were seeded into 24-well CytoView MEA plates (Axion Biosystems, # M384-tMEA-24W) containing 16-electrodes per well and media change was performed every 3–4 days.

MEA recordings were performed with the Maestro recording system using the AxIS suite software (Axion Biosystems Inc.) for data acquisition, off-line processing, and data analysis. Recordings for all patient lines began on Day 11 and were taken every 2–3 days. MEA plates were first equilibrated for 10-min in the Maestro at 37 °C and 5% CO_2_ before starting the recording. AxIS Navigator was used to obtain 10-min recordings in the “Neuronal Real-Time” and “Spontaneous” configuration. RAW recording files were processed offline in AxIS Navigator using a 6X standard deviation adaptive threshold for spike detection. Output SPK files were then processed with Axion Neural Metric Tool using the following settings: Active Electrode Criterion: ≥5 spikes/minute; Single Electrode Bursts: ISI Threshold, Minium number of spikes: 5, Maximum ISI: 100 ms; Network Bursts: Envelope, Threshold Factor: 2.5, Minium IBI 100 ms, Burst Inclusion: 50%, Minium number of electrodes: 75%; Synchrony Window: 20 ms. Wells that had <8 active electrode were excluded from analysis. CSV output files from each recording were loaded into the AxIS Metric Plotting tool and organized by cell line and day of the recording. Output files containing the raw MEA parameter values were analyzed using GraphPad Prism (GraphPad Software) and R.

### Statistical analysis and data visualization

All statistical analyses were conducted in R using established packages for differential expression, visualization, and enrichment analysis. Unless otherwise stated, statistical significance was defined as an adjusted p-value <0.05. Data visualizations, including PCA plots, volcano plots, correlation plots, and bar graphs, were generated using ggplot2 and related visualization packages.

## Results

### Neuronal phenotype context in NGLY1-deficient neurons

Immunofluorescence analysis at DIV28 revealed extensive MAP2-positive dendritic processes and the formation of interconnected neuronal networks across all glutamatergic neuronal cell lines (Figure 1). Control cultures exhibited dense MAP2-positive neuronal networks with widespread dendritic processes and punctate vGLUT2 staining. Neurons derived from the NGLY1 disease lines, 594D, 519D and 592C lines exhibited qualitative differences in network morphology, with greater cellular clustering and a less even distribution of MAP2-positive processes across the field. These observations indicate that while NGLY1-deficient iPSCs are capable of neuronal differentiation, mutation-dependent alterations in neuronal morphology and network organization are already apparent (by DIV28).

**FIGURE 1:**
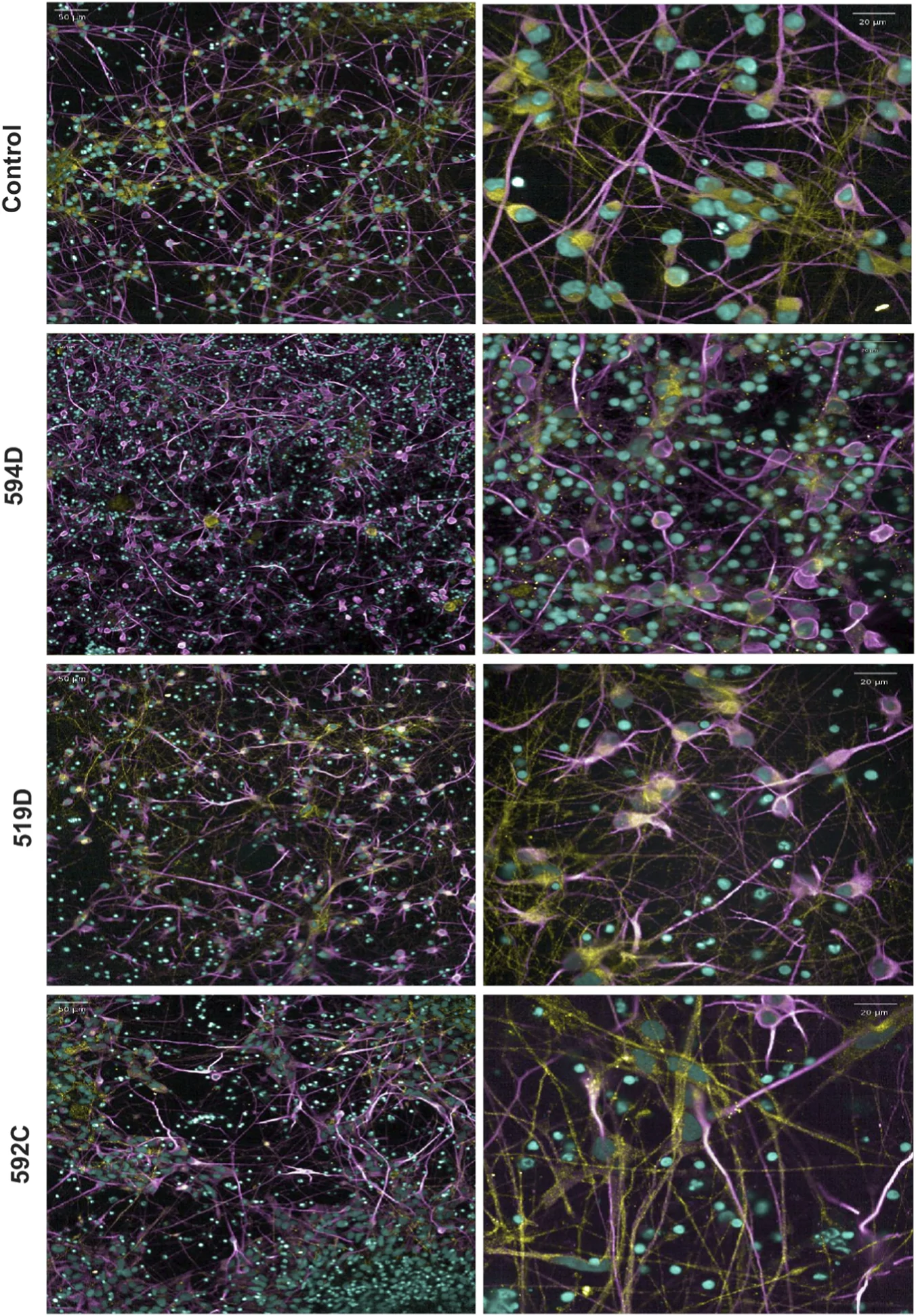
Representative images of neurons at day 28 *in vitro*. Images were acquired with ×20 (left) and ×63 (right) water objectives. Magenta: MAP2 (pan neuronal marker); turquoise: Hoechst (nuclear marker); yellow: vGLUT2 (vesicular glutamate transporter-2).

### Multi-omics expression profiling distinguishes disease cell lines from controls

To determine whether the morphological differences observed at DIV28 were accompanied by molecular alterations, we profiled the transcriptome and proteome of neurons derived from the three NGLY1 disease lines (594D, 519D, 592C) and the two controls (Control 1, WC31; Control 2, BD3). Principal component analysis of both RNA-seq and proteomics data cleanly separated the three disease lines from the pooled controls, with control replicates clustering tightly together and disease lines 592C and 594D occupying distinct regions of the PCA space (Figures 2A,B). Hierarchical clustering was concordant, grouping replicates by cell line and separating controls from disease lines in both datasets (Figures 2C,D).

**FIGURE 2:**
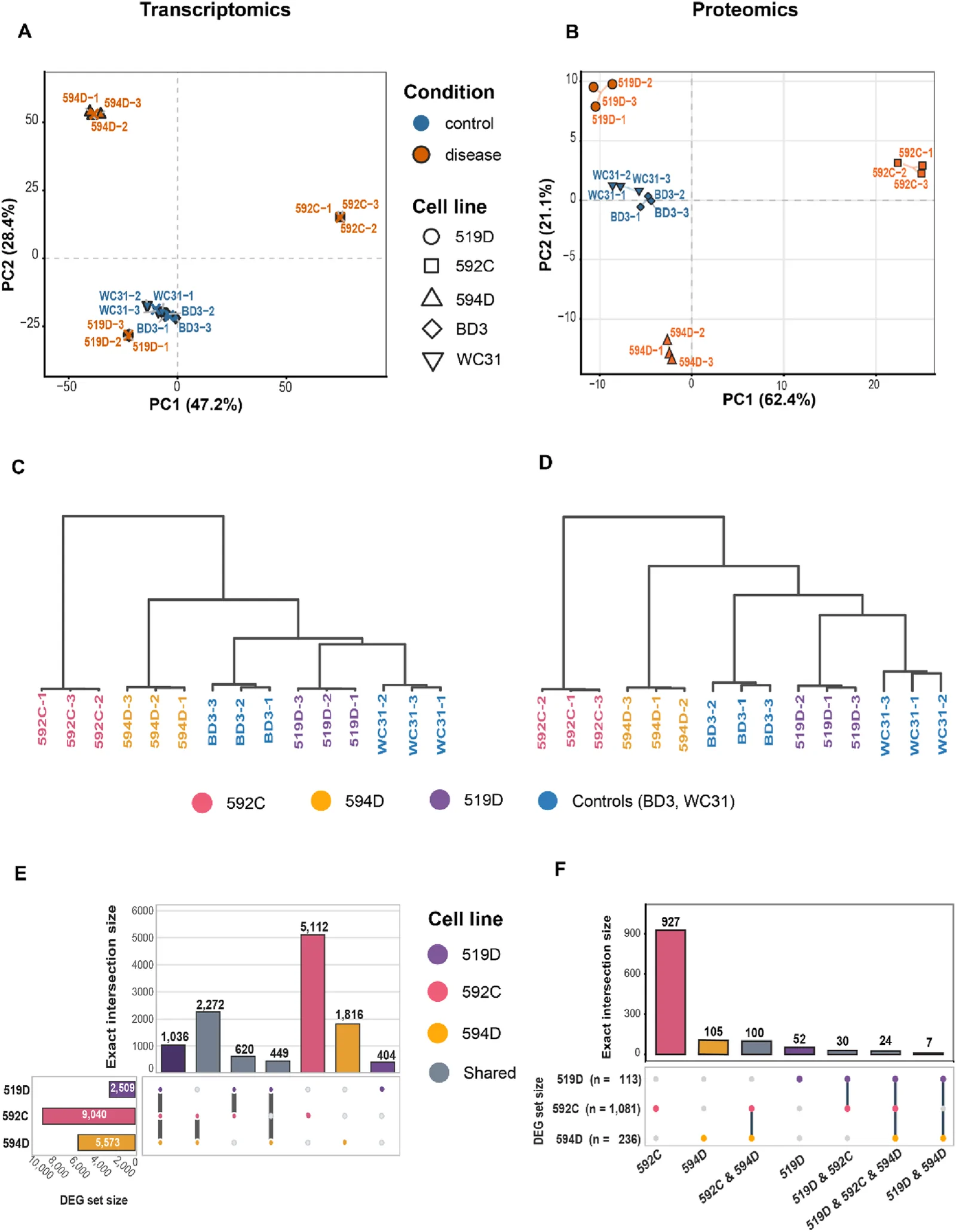
Transcriptomic and proteomic profiling separates disease cell lines from controls and reveals cell line–specific differential expression. **(A,B)** Principal component analysis of **(A)** RNA-seq and **(B)** proteomics data, with each point representing a biological replicate (n = 3 per cell line). Point color denotes condition (control, blue; disease, orange) and shape denotes cell line. Disease lines 592C, 594D, and 519D separate from the pooled controls (BD3, WC31), with 592C and 594D occupying distinct regions of the PCA space in both datasets. Percent variance explained is indicated on each axis. **(C,D)** Hierarchical clustering dendrograms of **(C)** RNA-seq and **(D)** proteomics replicates, colored by cell line (592C, pink; 594D, orange; 519D, purple; controls BD3 and WC31, blue). Replicates cluster by cell line, and control replicates group together separately from disease lines. **(E)** UpSet plot of differentially expressed genes (DEGs) from RNA-seq across the three disease lines relative to pooled controls. Horizontal bars (bottom left) show total DEG set size per line (592C, 9,040; 594D, 5,573; 519D, 2,509); vertical bars show exact intersection sizes, with the largest single-line set unique to 592C (5,112) and 404 DEGs shared across all three lines. **(F)** UpSet plot of significant differentially abundant proteins across the three lines relative to pooled controls (592C, n = 1,081; 594D, n = 236; 519D, n = 113). The majority of proteomic changes are unique to 592C (927), with limited overlap between lines and only seven proteins shared across all three.

Differential expression analysis relative to pooled controls revealed a graded response across lines, with 592C consistently the most affected: at the transcriptomic level, 592C, 594D, and 519D yielded 9,040, 5,573, and 2,509 differentially expressed genes (DEGs), respectively (Figure 2E), while at the proteomic level, using more conservative thresholds (FDR <0.05 and |log_2_FC| ≥ 0.585), they yielded 1,081, 236, and 113 significant proteins (Figure 2F). These changes were predominantly line-specific in both datasets: the largest DEG intersection was unique to 592C (5,112), with only 404 DEGs shared across all three lines, and 927 proteins were altered uniquely in 592C against just 7 shared across all lines.

Volcano plots resolved the direction and magnitude of these changes (Figure 3): 592C showed extensive, roughly balanced transcriptomic dysregulation (4,345 up, 4,695 down), 594D was skewed toward downregulation (2,082 up, 3,491 down), and 519D showed a more modest, upregulation-skewed response (1,421 up, 1,088 down), with the same rank order and directional consistency reflected at the protein level (592C: 503 up, 578 down; 594D: 111 up, 125 down; 519D: 81 up, 32 down). Consistent with the underlying genotype, *NGLY1* was significantly downregulated at the transcript level across all three disease lines, with a corresponding reduction in NGLY1 protein (Figure 3).

**FIGURE 3:**
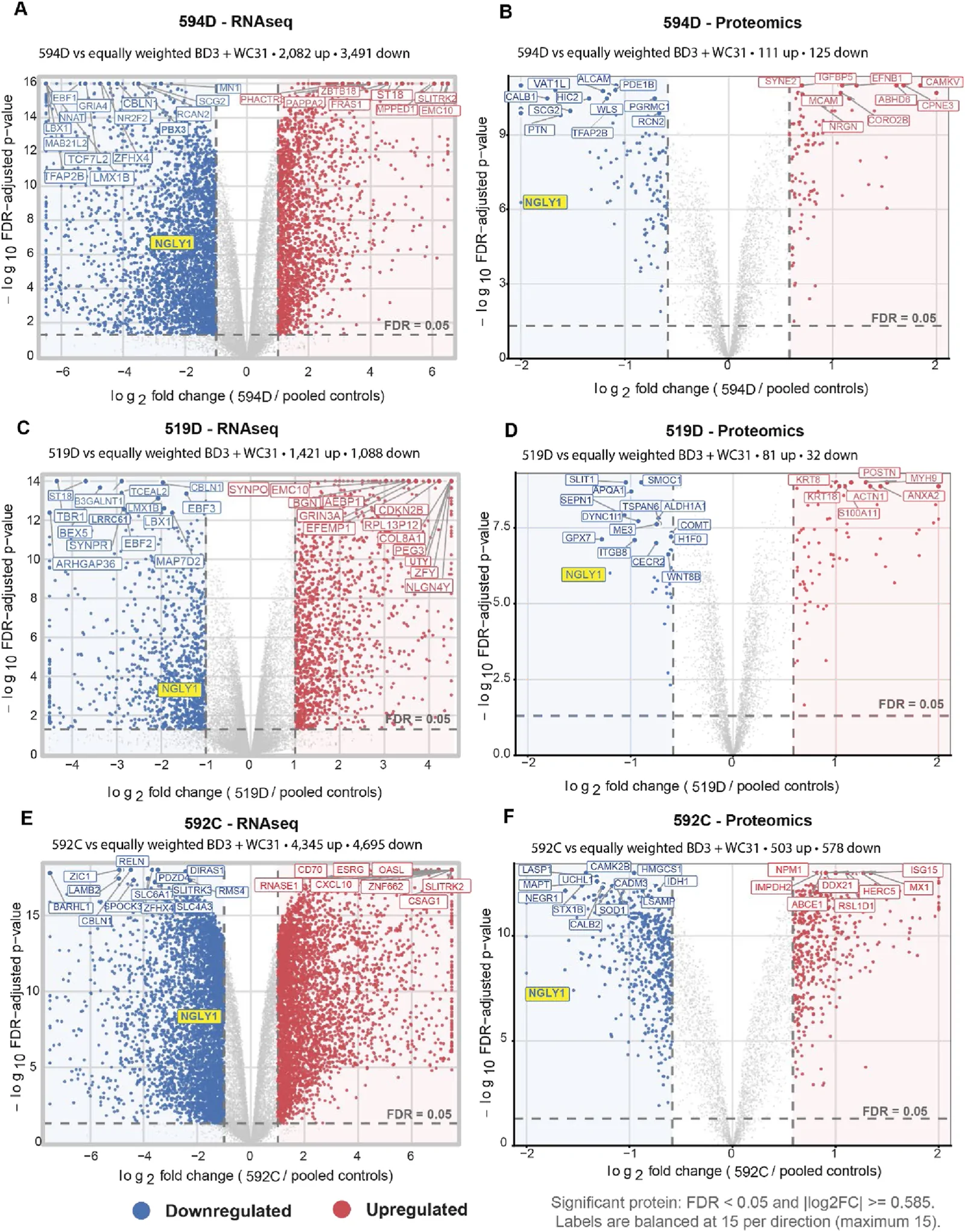
Volcano plots of differential expression at the transcriptomic and proteomic level for each disease cell line *versus* controls. Each row shows one disease cell line compared against the equally weighted controls (Control 1, WC31; Control 2, BD3): **(A,B)** 594D, **(C,D)** 519D, and **(E,F)** 592C, with RNA-seq (left column: **A,C,E**) and proteomics (right column: **B,D,F**). In each plot, log_2_ fold change (disease/pooled controls) is plotted against–log_10_ FDR-adjusted p-value. Significantly downregulated features are shown in blue and upregulated features in red; non-significant features are grey. The horizontal dashed line marks the significance threshold (FDR = 0.05). For RNA-seq, DEG counts are: 594D, 2,082 up/3,491 down **(A)**; 519D, 1,421 up/1,088 down **(C)**; 592C, 4,345 up/4,695 down. **(E)** For proteomics, significant proteins (FDR <0.05 and |log_2_FC| ≥ 0.585) are: 594D, 111 up/125 down **(B)**; 519D, 81 up/32 down **(D)**; 592C, 503 up/578 down. **(F)** The top features per direction are labeled (up to 15 per direction). *NGLY1* (RNA-seq) and NGLY1 (proteomics) are highlighted in yellow in all panels.

### Transcriptional dysregulation is substantially attenuated at the protein level

To assess whether transcriptomic changes were propagated to the protein level, we restricted both assays to the 4,128 gene products detected in common and compared their behavior across the three disease lines. Most jointly tested gene products fell into the neither category, indicating no significant change in either assay, ranging from 95.5% in 519D to 87.5% in 594D and 58.5% in 592C, consistent with 592C being the most extensively dysregulated line (Figure 4A). Among features that did change, the largest classes reflected regulation detected at the RNA level alone. In 592C, 14.3% of gene products were altered at the transcript level while remaining equivalent or stable at the protein level, alongside a smaller protein only fraction (12.0%) and a concordant significant class comprising 12.4% of tested products. Truly discordant significant changes, in which RNA and protein moved in opposite directions, were rare in all lines (Figure 4A).

**FIGURE 4:**
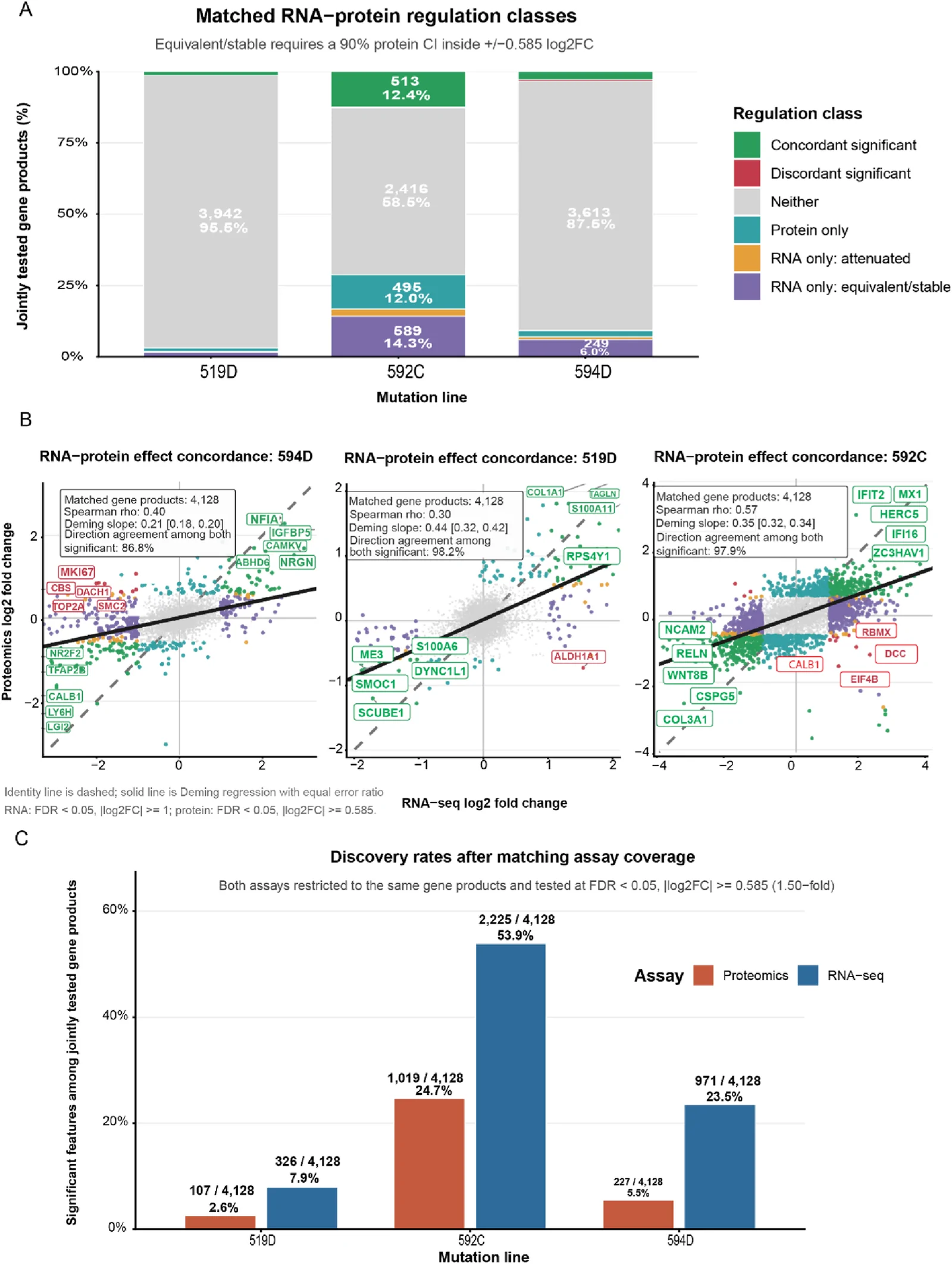
Transcriptional changes are systematically attenuated at the protein level with concordant directionality across NGLY1 disease lines. Analyses were restricted to the 4,128 gene products detected in both the RNA-seq and proteomics assays. **(A)** Stacked bar plot showing the distribution of matched RNA-protein regulation classes for each disease line (519D, 592C, 594D), expressed as a percentage of jointly tested gene products. Classes comprise concordant significant, discordant significant, neither, protein only, RNA only attenuated, and RNA only equivalent or stable, the last defined by a 90% protein confidence interval falling within ± 0.585 log_2_FC. Most gene products showed no significant change in either assay (neither class), with the largest regulated fractions in 592C. **(B)** Scatter plots of proteomics *versus* RNA-seq log_2_ fold change for matched gene products in 594D, 519D, and 592C. The dashed line denotes identity and the solid line denotes Deming regression assuming an equal error ratio. Spearman correlation, Deming slope with 95% confidence interval, and direction agreement among features significant in both assays are indicated for each line. Selected gene products are labelled. Significance thresholds were FDR <0.05 and |log_2_FC| ≥ 1 for RNA and FDR <0.05 and |log_2_FC| ≥ 0.585 for protein. **(C)** Grouped bar plot showing the proportion of jointly tested gene products called significant by each assay when both were evaluated under identical thresholds (FDR <0.05, |log_2_FC| ≥ 0.585, equivalent to 1.50-fold). RNA-seq recovered more significant features than proteomics in every line, with the largest difference in 592C.

Direct comparison of matched effect sizes confirmed a positive but modest quantitative relationship between the two assays (Figure 4B). Spearman correlations increased from 519D (rho = 0.30) to 594D (rho = 0.40) and 592C (rho = 0.57), and Deming regression slopes below unity in every line (0.21–0.44) indicated that protein fold changes were consistently compressed relative to their transcript counterparts. Despite this attenuation, the direction of change agreed strongly among features significant in both assays, reaching 86.8% in 594D and exceeding 97% in both 519D and 592C. When both assays were tested under identical thresholds (FDR <0.05, |log_2_FC| ≥ 0.585), RNA-seq recovered more significant features than proteomics in every line, most markedly in 592C (53.9% *versus* 24.7%), reflecting the greater dynamic range and sensitivity of the transcriptomic assay under matched coverage (Figure 4C).

This discovery gap was robust and did not arise solely from the choice of significance thresholds, persisting across a range of FDR levels and effect-size cutoffs with RNA-seq consistently recovering more significant features than proteomics (Supplementary Figure S1A). Tracing the fate of significant RNA changes through the proteomics workflow showed that the large majority of RNA DEGs lacked a confidently measured cognate protein, most often because no human protein was identified, with concordant and discordant significant protein outcomes each accounting for only a small fraction (Supplementary Figure S1B). Among transcripts that were quantified at the protein level, many with large RNA fold changes had protein estimates whose 95% confidence intervals fell within or near the ± 0.585 log_2_FC equivalence region, indicating genuine dampening of the corresponding protein response (Supplementary Figure S1C). Consistent with this, a simulation in which protein effect sizes were set equal to the observed RNA effects predicted substantially more differentially abundant proteins than were actually observed, so the shortfall in protein-level discoveries cannot be explained by statistical power alone (Supplementary Figure S1D). Finally, proteomic coverage increased monotonically with transcript abundance, such that low-abundance transcripts were markedly under-represented among quantified proteins (Supplementary Figure S1E). Together these results indicate that transcriptional dysregulation in NGLY1-deficient neurons is only partially reflected at the protein level, owing both to genuine attenuation of protein effect sizes and to reduced proteomic sensitivity for low-abundance gene products, while the direction of change remains highly concordant where both assays detect a signal.

### Differential exon usage marks discordant genes and implicates NGLY1 loss in splicing dysregulation

To ask whether the RNA-protein discordance was associated with altered transcript structure, we tested for differential exon usage (DEU) with DEXSeq across the matched gene set. Strongly discordant genes were enriched for significant DEU relative to other matched, DEXSeq-tested genes in a line-dependent manner. Among genes showing strong quantitative discordance, DEU enrichment reached an odds ratio of 2.05 in 519D (q = 0.053) but was absent in 592C (OR 1.01) and, if anything, depleted in 594D (OR 0.58, q = 6.5e-04) (Figures 5A,B). For genes whose protein change was not matched at the RNA level, 519D again showed the strongest enrichment (OR 2.82, q = 0.004), with weaker and nonsignificant effects in 592C and 594D (Figure 5B). Representative discordant genes carrying gene-level DEU included extracellular matrix and collagen genes (COL1A1, COL1A2, COL12A1, COL4A1, COL18A1) prominent in 592C, together with CBS in 519D and GRIA4 and MEIS1 in 594D, spanning a wide range of protein minus RNA effect differences (Figure 5C).

**FIGURE 5:**
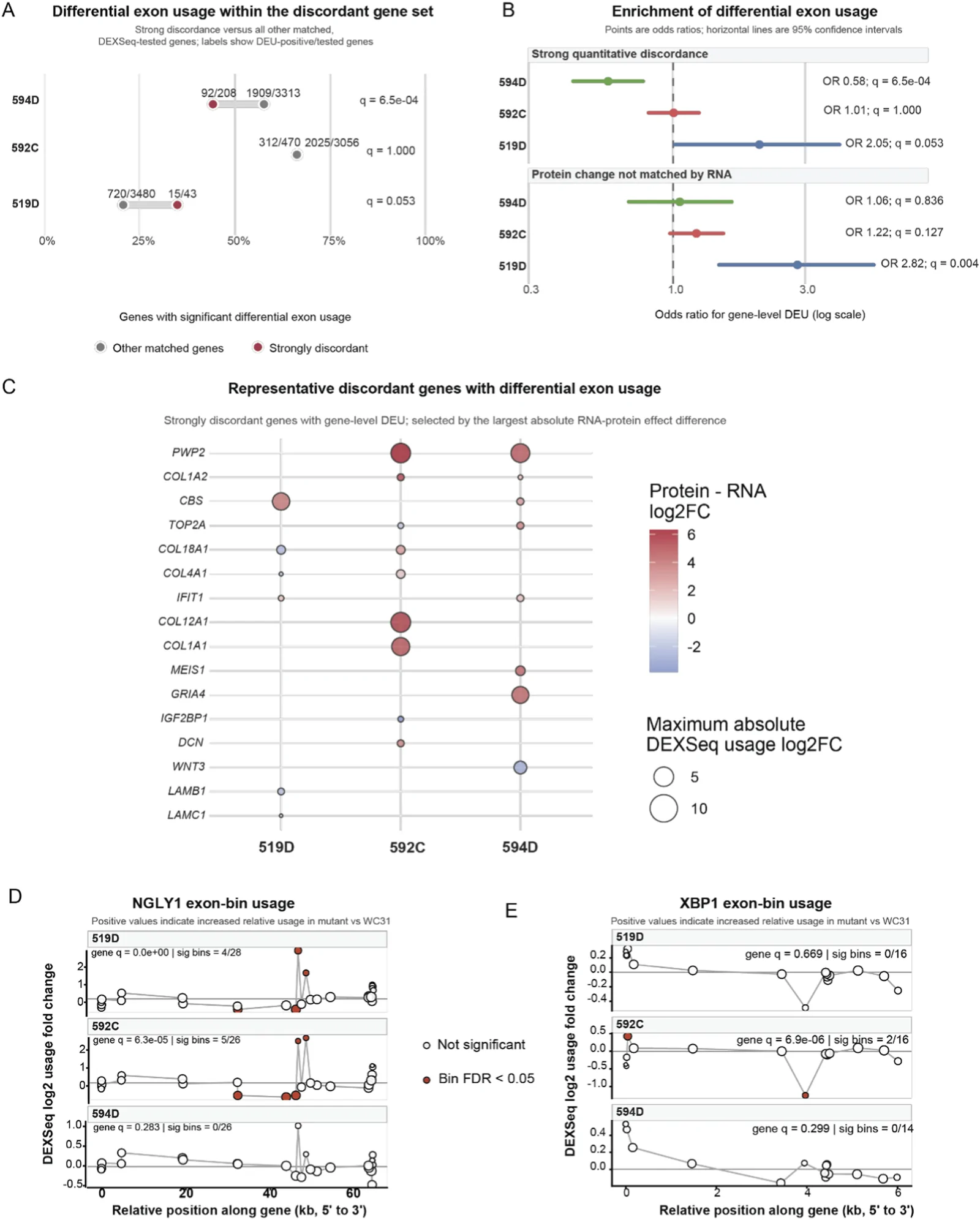
Differential exon usage is enriched among discordant genes and affects NGLY1 and XBP1 in a line-specific manner. **(A)** Proportion of genes with significant differential exon usage (DEU) among strongly discordant genes compared with all other matched, DEXSeq-tested genes, shown for each disease line (519D, 592C, 594D). Labels give the number of DEU-positive genes over the number tested, with q-values from the corresponding enrichment test. **(B)** Odds ratios for gene-level DEU in two discordance categories, strong quantitative discordance and protein change not matched by RNA, plotted on a log scale with 95% confidence intervals and q-values. Enrichment was strongest in 519D. **(C)** Representative strongly discordant genes with gene-level DEU, selected by the largest absolute RNA-protein effect difference. Point colour encodes the protein minus RNA log_2_ fold change and point size encodes the maximum absolute DEXSeq usage log_2_ fold change, shown per line. **(D)** NGLY1 exon-bin usage along the transcript (5′to 3′) for each line. Points show DEXSeq log_2_ usage fold change relative to control 1 (WC31), with positive values indicating increased relative usage in mutant, and filled points denoting bins at FDR <0.05. Gene-level q-values and the number of significant bins are annotated per line. **(E)** XBP1 exon-bin usage, plotted as in **(D)**.

Because NGLY1 loss is expected to perturb transcript processing, we examined exon-bin usage at NGLY1 itself and at the canonical stress-response regulator XBP1. NGLY1 showed significant gene-level DEU in 519D and 592C, with several significant exon bins concentrated toward the 3′region of the transcript, whereas 594D did not reach gene-level significance (Figure 5D). XBP1 showed a focal, significant usage change in 592C (2 significant bins, q = 6.9e-06) but not in 519D or 594D (Figure 5E), consistent with line-specific engagement of the unfolded protein response.

At the global level, the burden of differential exon usage mirrored the overall transcriptomic response, with 592C carrying the largest number of significant genes (7,332) and exon bins (49,752), followed by 594D (6,027 genes, 33,282 bins) and 519D (2,253 genes, 8,502 bins) (Supplementary Figure S2A). DEU genes were substantially shared between the two most affected lines, with 2,357 genes common to 592C and 594D and 1,031 shared across all three, alongside large line-specific sets in 592C (2,558) and 594D (1,460) (Supplementary Figure S2B). A targeted survey of NGLY1, XBP1 and ERN1 together with UPR and ER-proteostasis effectors (including HSPA5, DDIT3, HSP90B1, HERPUD1, CANX and CALR) and the core nonsense-mediated decay machinery (UPF1 to UPF3B and SMG1 to SMG7) showed that these regulators were themselves subject to significant exon-usage changes, most extensively in 592C, indicating that NGLY1 deficiency is accompanied by remodelling of the very pathways responsible for ER quality control and transcript surveillance (Supplementary Figure S2C).

### Pathway analysis reveals convergent extracellular matrix remodeling alongside line-specific neuronal and translational programs

To move from individual genes to coordinated biological programs, we tested curated pathway sets across RNA-seq, proteomics, and the concordant marker set for each disease line, selecting the strongest directional term within each program (Figure 6). A subset of programs was dysregulated across all three lines, most prominently extracellular matrix, collagen and adhesion, which was significant in every line and every assay layer and was among the few programs to reach concordant significance in 519D. Muscle and contractile differentiation, ion transport and membrane potential, GPCR and receptor signaling, and development and morphogenesis were also broadly affected, though these programs frequently diverged in direction between lines rather than moving uniformly (Figure 6). This direction divergence, in which the same program was activated in one line and repressed in another, was a defining feature of the dataset and distinguished the shared core from truly convergent regulation.

**FIGURE 6:**
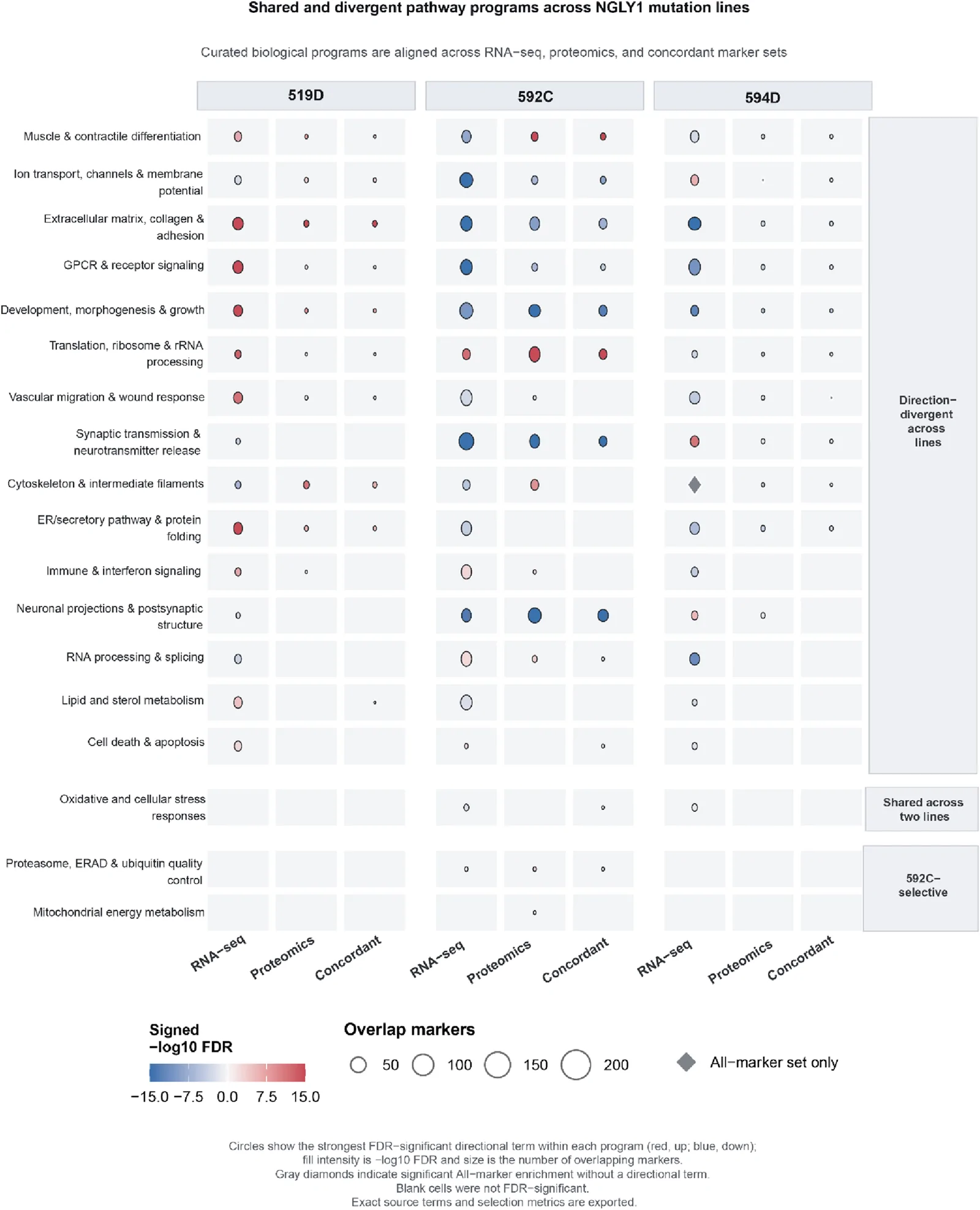
Curated biological programs reveal a shared extracellular matrix core and line-specific neuronal and translational reprogramming across NGLY1 mutation lines. For each disease line (519D, 592C, 594D) and each analysis layer (RNA-seq, proteomics, concordant marker set), the strongest FDR-significant directional term within each curated program is shown. Circle color encodes the signed -log_10_ FDR (red, up; blue, down), fill intensity encodes the magnitude of -log_10_ FDR, and circle size encodes the number of overlapping markers. Grey diamonds indicate significant all-marker enrichment without a directional term, and blank cells were not FDR-significant. Programs are grouped at right into those that were direction-divergent across lines, those shared across two lines, and those selective to 592C.

Beyond this shared core, the lines showed distinct dominant programs. 592C was by far the most transcriptionally and translationally engaged line, uniquely reaching significance for proteasome, ERAD and ubiquitin quality control and for mitochondrial energy metabolism, and showing the strongest signals in translation, ribosome and rRNA processing, synaptic transmission, and neuronal projection and postsynaptic structure, the last with clear concordant support across assays (Figure 6). Oxidative and cellular stress responses were shared between 592C and 594D but absent in 519D. These patterns indicate that, while all three lines converge on matrix and adhesion remodeling, each engages a partly private downstream program.

The per-line cross-omics analyses reinforced these conclusions and resolved the direction of individual pathways (Supplementary Figures S3–S5). In 519D, enrichment was dominated by extracellular matrix organization, collagen formation and ECM proteoglycans, with upregulated matrix structural constituents at both the transcript and protein level and a set of downregulated cell-cycle and chromosome-segregation terms confined to RNA-seq (Supplementary Figure S3). In 592C, the analysis revealed a striking split between strongly upregulated translation machinery, including cytoplasmic translation, rRNA processing, ribosome biogenesis and peptide chain elongation with consistent concordant support, and broadly downregulated neuronal programs such as chemical synaptic transmission, synapse assembly and regulation of membrane potential (Supplementary Figure S4). In 594D, downregulated terms centred on trans-synaptic signalling, neurotransmitter receptor activity, GPCR signalling and neuronal system pathways, alongside extracellular matrix and developmental lineage programs, with much of the signal carried by RNA-seq and only partial proteomic corroboration (Supplementary Figure S5). Together these results show that NGLY1 deficiency converges on extracellular matrix remodelling across genetic backgrounds while producing line-specific reprogramming of translation, synaptic and developmental pathways, with the strongest and most cross-validated program shifts occurring in 592C.

### Integrating concordant markers with pathways links molecular signatures to the NGLY1 clinical phenotype

To connect the molecular findings to the clinical presentation of NGLY1 deficiency, we constructed a network linking concordant RNA and protein markers to their enriched pathways and, in turn, to the documented symptom domains of the disorder (Figure 7). Markers were ranked by evidence and classified as common to all three lines, pairwise shared, or line-specific. A core set of markers was altered concordantly across all three lines, including secreted and matrix-associated factors such as CXCL14, PTN, S100A6, GPNMB, SORCS2, SFRP2 and MTTP, several with upregulated direction across backgrounds. Around this shared core, each line contributed its own set of specific markers, for example RELN, ISG15, CAMK2B, MAPT and CSPG5 among the 592C-specific set and TFAP2B, NFIA, PLCB1 and NRGN among those specific to 594D (Figure 7).

**FIGURE 7:**
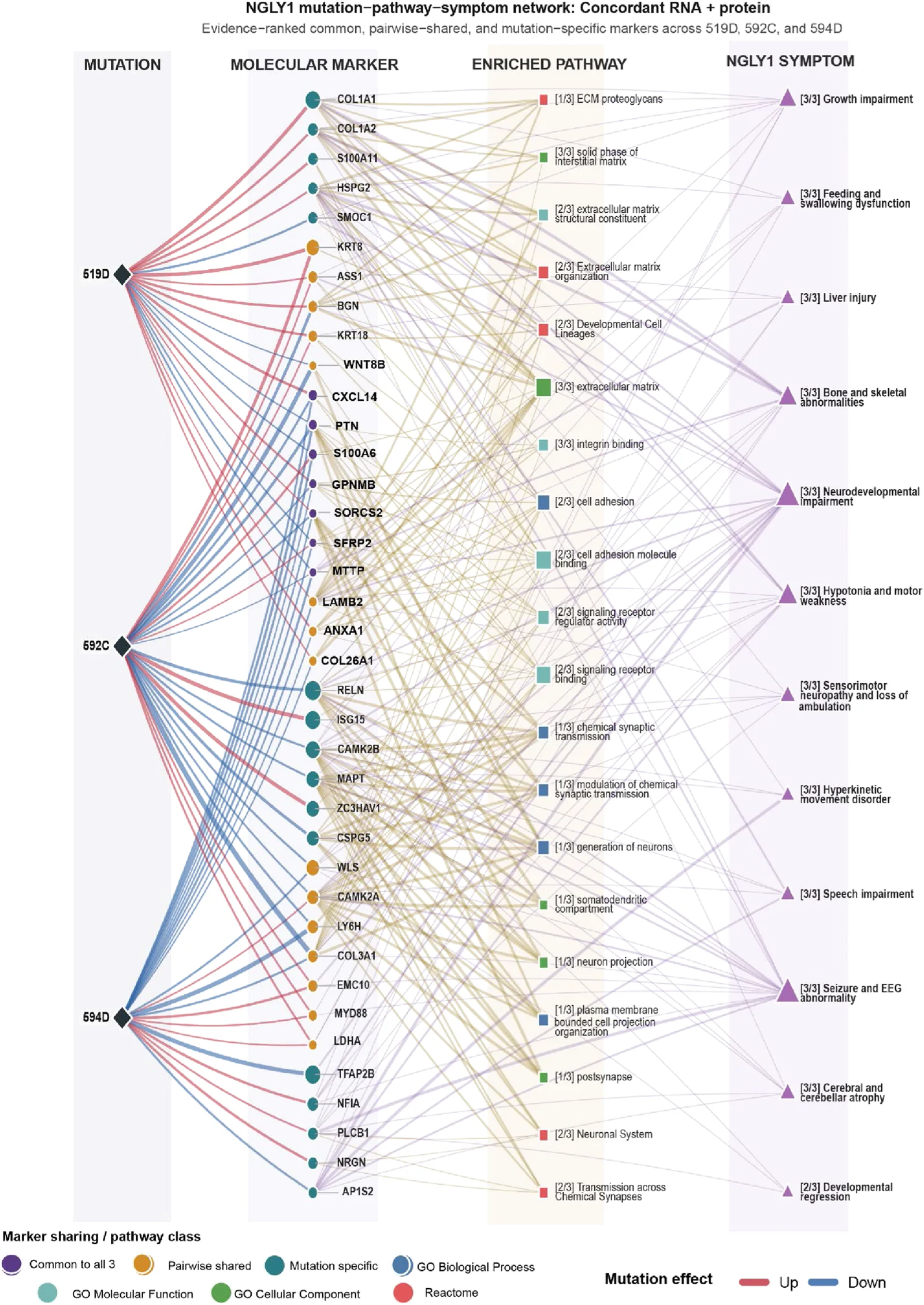
A concordant molecular marker to pathway to symptom network links NGLY1 cell-line signatures to the clinical phenotype. Four-column network built from concordant RNA and protein markers across the three disease lines (519D, 592C, 594D). The first column shows mutation lines, the second shows molecular markers colored by sharing class (common to all three, pairwise shared, or line-specific), the third shows enriched pathways colored by database (GO Biological Process, GO Molecular Function, GO Cellular Component, and Reactome) with bracketed fractions indicating how many lines contributed to each term, and the fourth shows NGLY1 symptom domains with bracketed fractions indicating cross-line support. Edges between mutation lines and markers are colored by the direction of the concordant effect (red, up; blue, down), and node size for symptoms reflects the strength of support. Markers, pathways, and symptoms are evidence-ranked, and edges denote membership or documented association.

At the pathway level, the most broadly supported terms were again matrix-related, with extracellular matrix, solid phase of interstitial matrix and integrin binding drawing markers from all three lines, alongside adhesion and signalling receptor programs shared by two lines and synaptic terms contributed largely by single lines (Figure 7). Crucially, these pathways connected to symptom domains that recapitulate the human NGLY1 phenotype. Programs affected across all three lines mapped onto the most penetrant clinical features, including growth impairment, feeding and swallowing dysfunction, liver injury, bone and skeletal abnormalities, neurodevelopmental impairment, hypotonia and motor weakness, sensorimotor neuropathy, hyperkinetic movement disorder, speech impairment, seizure and EEG abnormality, and cerebral and cerebellar atrophy, while developmental regression was supported by two lines (Figure 7). This integration shows that the convergent matrix and adhesion signature and the line-specific synaptic and neuronal programs together plausibly account for the multisystem symptom spectrum of NGLY1 deficiency, providing a molecular framework that links the *in vitro* signatures back to patient phenotypes.

### Functional analysis reveals altered neuronal network activity in NGLY1 deficient neurons

We next set out to examine the functional consequences of NGLY1 deficiency in each patient’s neuronal line using MEAs. Previous studies using manual patch-clamp found NGLY1 mutations can either increase or decrease the apparent action potential threshold and magnitude of glutamatergic neurons, suggesting a mix of hypoexcitable or hyperexcitable phenotypic changes depending on the specific genotype (Manole et al., 2023). Unlike the terminal nature of manual patch-clamp measurements, MEA recordings are non-invasive and permit multiple measurements, allowing neuronal network maturation to be tracked throughout the culture. All neuronal lines exhibited spontaneous activity detected on DIV11 that increased through DIV29 (Supplementary Figure S6). Control cultures displayed progressive increases in number of active electrodes, weighted mean firing rate (wMFR), and both individual and network bursting, reflecting neuronal network maturation *in vitro* (Figures 8A,B). In contrast, NGLY1-deficient neurons exhibited reduced MFR, a slower progressive increase in the number of active electrodes, delayed onset of bursting, and altered network bursting parameters (Figures 8A,B). Raster plots from DIV29 recordings illustrate altered synchronization and network activity across all NGYL1-deficient lines (Figures 8C,D).

**FIGURE 8:**
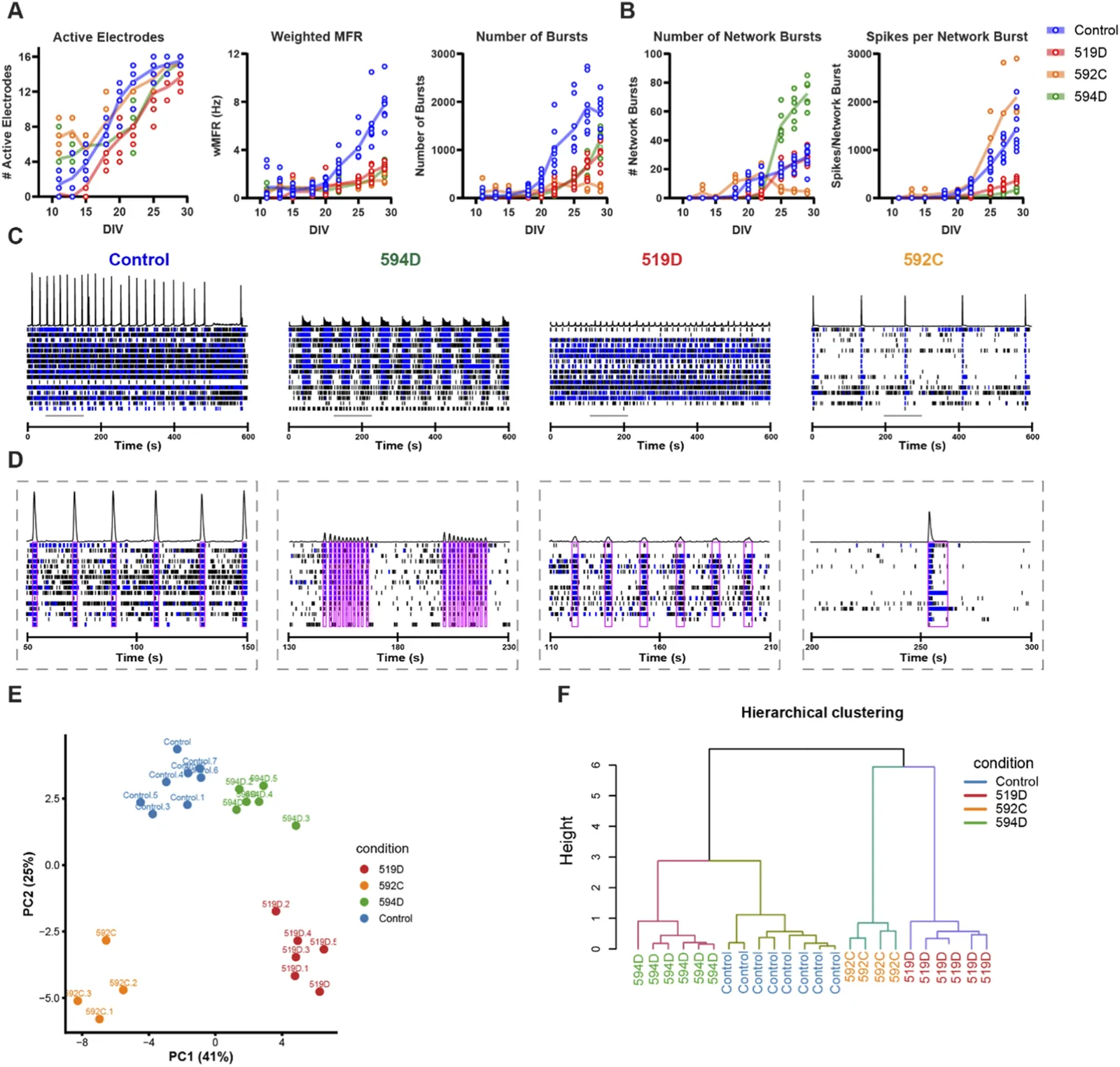
Multi-electrode array analysis reveals altered neuronal network activity in NGLY1-deficient neurons. **(A,B)** Longitudinal assessment of neuronal network development in control (blue) and NGLY1-deficient lines (594D (green), 519D (red), 592C (yellow)). Metrics shown include number of active electrodes, weighted mean firing rate, number of bursts, number of network bursts, and spikes per network burst measured across DIV11-DIV29. **(C)** Representative raster plots of spontaneous neuronal activity for each line at DIV29, illustrating differences in bursting and network firing patterns. **(D)** Expanded views of network burst events highlighting differences in burst structure, timing, and synchronization between control and NGLY1-deficient neurons. **(E)** Principal component analysis of electrophysiological features demonstrating separation between NGLY1-deficient and control lines, indicating distinct functional network states. **(F)** Hierarchical clustering of electrophysiological profiles across conditions, separating control and NGLY1-deficient neurons.

Similar to previous reports, differences in neuronal function were genotype specific and these changes could be indicative of either a hypoexcitable or hyperexcitable phenotype. For example, 594D neurons displayed increased number of network bursts over control neurons suggesting a hyperexcitable phenotype (Figure 8B). However, a smaller number of spikes per network burst suggests weaker network strength and a hypoexcitable phenotype. PCA of 49 MEA parameters from all DIV29 recordings show distinct clustering of each patient line (Figure 8E). Similar to the clustering seen in the PCA of the RNA-seq and proteomics data (Figures 2A,B), each neuronal patient line forms non-overlapping clusters and suggests a functional connection with the transcriptomics and proteomics consequences of each individual genotype.

## Discussion

This study reframes NGLY1 deficiency as a set of genotype-specific molecular states rather than a single, uniform neuronal phenotype. By comparing three distinct patient genotype head-to-head within one neuronal system, we found that transcriptomic and proteomic profiles segregate cleanly by genotype, that only a small molecular core is shared across all three lines, and that overlapping clinical phenotypes are reached through distinct pathway routes. These genotypes nonetheless converge on a common functional endpoint of impaired network maturation, indicating that shared clinical features can emerge from divergent molecular trajectories. This design carries an inherent and important constraint that frames the interpretation of all downstream results. With a single patient line per genotype, genotype and genetic background are not separable, and the study does not include isogenic gene-corrected controls; we therefore treat the findings as discovery stage and hypothesis-generating rather than a definitive attribution of specific features to NGLY1 loss. This constraint is intrinsic to comparative work in an ultra-rare disorder, where patient material is fundamentally limited. Importantly, our objective, resolving between-genotype convergence *versus* divergence, is complementary to, rather than a substitute for, rescue-based designs that isolate NGLY1-attributable effects within a fixed background (Manole et al., 2023). Comparable multi-omics studies of patient-derived neurons in other rare neurological disorders have likewise shown that integrating several heterogeneous lines across omics layers yields robust, network-level disease signatures despite small sample numbers (Li et al., 2021). Read in this light, the genotype-resolved architecture we describe is best interpreted as a prioritized map of candidate molecular routes requiring isogenic validation, which we identify below as the essential next step.

A major finding is that the shared molecular core is limited, while genotype-specific signatures are substantially expanded. Recurrent cross-omics features, most notably the concordant reduction of NGLY1 itself, define a small conserved set, whereas the majority of markers and pathways show line-dependent enrichment. This architecture has practical consequences for how molecular features should be interpreted and prioritized: candidates that satisfy multiple criteria, reproducibility across biological replicates, relevance to NGLY1 biology, cross-layer support across omics modalities, and linkage to disease-relevant neuronal function, represent the most robust starting points, whether the goal is mechanistic subgrouping, model selection, or eventual biomarker development. Applying these criteria distinguishes a small set of shared, high-confidence features from a larger tier of genotype-weighted candidates appropriate for targeted follow-up.

A plausible interpretation is therefore that transcriptional dysregulation in NGLY1-deficient neurons is partially buffered at the protein level by homeostatic control of protein abundance, a notable observation given the central role of NGLY1 in protein quality control.

Cross-omics integration further refines biomarker prioritization. RNA–protein concordance is positive but moderate and its strength varies by genotype, indicating mutation-dependent coupling between transcript and protein abundance. Importantly, the roughly twenty-to-fifty-fold difference between the number of differentially expressed genes and differentially abundant proteins does not reflect a single cause. Transcriptional changes do not propagate uniformly to the proteome; post-transcriptional regulation and differences in protein stability contribute substantially to this divergence. Part of the gap is technical; proteomic coverage were markedly under-represented among quantified proteins, and the majority of RNA differentially expressed genes had no confidently identified cognate protein (Supplementary Figure S1). However, the gap is not purely a coverage artifact. Among transcripts that were quantified at the protein level, effect sizes were systematically compressed relative to the transcriptome, and a simulation in which protein effects were set equal to the observed RNA effects predicted far more differentially abundant proteins than were observed, indicating genuine post-transcriptional attenuation beyond limited statistical power (Supplementary Figure S1). This is mechanistically coherent with NGLY1 biology, since NGLY1 loss impairs ERAD and NFE2L1-mediated proteosome regulation and thereby alters protein turnover independently of transcription (Tomlin et al., 2017; Forcina et al., 2022). A plausible interpretation is therefore that transcriptional dysregulation in NGLY1-deficient neurons is partially buffered at the protein level by homeostatic control of protein abundance, a notable observation given the central role of NGLY1 in protein quality control.

A second, non-mutually-exclusive contributor to this discordance is regulation at the level of transcript structure. Differential exon usage was widespread in all three lines and, critically, was enriched among RNA to protein discordant genes in a mutation-dependent manner, most strongly 592C (Figure 5). Coordinated exon-usage changes were evident across canonical unfolded protein response and ER-proteostasis effectors and across nonsense-mediated decay machinery, and NGLY1 itself showed significant differential exon usage in 519D and 592C. Because gene-level RNA-seq quantification aggregates reads across isoforms, a shift toward nonproductive or nonsense-mediated decay-targeted isoforms can reduce protein output without a proportional change in total transcript signal, offering a direct mechanism for a subset of the observed discordance. We interpret this conservatively, since the enrichment was strongest in 592C and weaker or absent in 594D, and since exon-level testing is highly sensitive against a high global background of exon-usage changes. We also note that the IRE1 mediated XBP1 splicing event involves a 26-nucleotide unconventional intron below the resolution of exon-bin counting, so our detection of an exon-usage signal at XBP1 in 592C is suggestive rather than definitive and would require targeted quantification of the spliced to un-spliced ratio to confirm.

The pathway analyses further reinforce a stratified biomarker model. Using over-representation analysis across matched RNA, protein and concordant marker sets, we found that although the three NGLY1 genotypes share core clinical features, their underlying molecular organization differs substantially. A single program, extracellular matrix, collagen and adhesion, was over-represented in every line and every assay layer and reached concordant significance even in the least affected line, 519D, marking it as the principal convergent axis of NGLY1 deficiency across genetic backgrounds (Figure 5; Supplementary Figures S3–S5). Beyond this shared core, most programs were engaged to markedly different extents and, frequently, in opposing directions across lines. Muscle and contractile differentiation, ion transport and membrane potential GPCR and receptor signaling, development and morphogenesis, and synaptic transmission were broadly affected but direction-divergent between lines rather than uniformly shifted, so the same program could be activated in one genotype and repressed in another. This direction divergence, rather than a shared directional signature, was a defining feature of the dataset and distinguishes convergent biology from genotype-specific reprogramming.

The line-resolved analyses further show that each genotype is dominated by a distinct downstream program. The 592C line was by far the most transcriptionally and translationally engaged line and was uniquely over-represented for proteosome, ERAD and ubiquitin quality control and for mitochondrial energy metabolism, alongside strongly upregulated translation, ribosome and rRNA processing terms with concordant RNA and protein support and broadly downregulated synaptic and neuronal programs (Figure 6; Supplementary Figure S4). The 594D line was intermediate and predominantly RNA-driven, with downregulation of trans-synaptic signaling, neurotransmitter receptor activity, GPCR signaling and neuronal system pathways together with extracellular matrix and developmental lineage programs (Figure 6; Supplementary Figure S5). The 519D showed the most restricted response, concentrated on extracellular matrix organization, collagen formation and matrix structural constituents with concordant support, together with a set of downregulated cell-cycle and chromosome-segregation terms confined to the transcript level (Figure 6; Supplementary Figure S3). This pattern supports two complementary interpretations of the genotype-resolved features. Shared mechanistic features, principally the concordant reduction of NGLY1 and the convergent extracellular matrix program, may serve as cross-genotype indicators of disease activity, whereas genotype-weighted programs are more informative for mechanistic subgrouping and for selecting appropriate readouts in patient-derived preclinical models. Either use ultimately depends on validation in matched genetic backgrounds.

Consistent with this stratified view, integrating the concordant markers with their enriched pathways and with Human Phenotype Ontology symptom modules linked the convergent matrix and adhesion signature and the line-specific synaptic and neuronal programs to the overlapping multi-system features of NGLY1 deficiency, illustrating how divergent molecular routes can converge on a shared clinical spectrum (Figure 7).

The MEA data adds an essential functional validation layer to this framework. Genotype-dependent changes in firing, bursting, and network activity proposes disrupted neuronal culture maturation across NGLY1-deficient lines relative to control culture, but potentially could result from lower neuronal viability of disease lines throughout the duration of the MEA culture. MEA cultures of NGLY1-deficient neuronal lines were maintained past DIV29 and displayed progressive increases in activity through DIV43 (Supplementary Figure S7). While the number of active electrodes plateaued and remained stable, the wMFR continued to increase during this period, suggesting differences in activity between disease and control lines are the result of altered functional maturation and not decreasing viability throughout the duration of the culture (Supplementary Figure S8).

Functional differences between control and NGLY1-deficient lines measured with MEAs could also arise from alterations in the excitatory and inhibitory neuronal balance of these cultures. Differentiation of iPSC into excitatory neurons can produce off-target inhibitory GABAergic neuronal populations (Strano et al., 2020). Curated examination of ligand-gated channels reveals distinct differences in the transcriptomic and proteomic profiles of both ionotropic glutamate receptors and GABA receptor (iGluR) and gamma-aminobutyric acid (GABA) receptor (GABAR) subunits (Supplementary Figure S9). Changes in iGluR and GABAR subunit expression across NGLY1-deficient lines could be responsible for the mix of hypoexcitable and hyperexcitable MEA parameter changes observed here and by others (Abbott et al., 2023). Alterations in ligand-gated ion channel expression can be compounded by additional changes in synaptic signaling, including deficits in GABA production as was previously reported in NGLY1-deficent midbrain organoids (Abbott et al., 2023). As each genotype has a unique signature of altered MEA parameters at DIV29 (Figure 8E) reflecting both hyperexcitable and hypoexcitable changes, functional biomarkers will likely require multiple endpoint measurements to capture all NGLY1 genotypes, requiring a panel-based approach that is more appropriate than a one-metric-fits-all model (Rosa et al., 2020).

These findings align with prior work documenting synaptic, proteostasis, mitochondrial, and maturation abnormalities in human neuronal models of NGLY1 deficiency. Manole et al. (2023) reported disruption of protein aggregate handling, mitochondrial homeostasis, and synaptic function in patient-derived cortical neurons, with rescue upon restoration of NGLY1 (Manole et al., 2023). Abbott et al. (2023) examined overlapping genotypes in midbrain organoid models and reported altered neuronal development characterized by reduced neuronal and glial markers, decreased GABA levels, and diminished tyrosine hydroxylase staining, consistent with dopaminergic pathway disruption (Abbott et al., 2023). The present study extends this literature by demonstrating substantial genotype dependence in cross-omics structure and functional network state, with only limited shared molecular features beyond NGLY1 itself. These findings have important implications for biomarker studies, as they provide a mechanistic explanation for why candidate markers identified in one model background may not generalize across the full disease spectrum. In other words, heterogeneous downstream biology may coexist with partially overlapping clinical phenotypes, and biomarker development should be designed with this genotype-dependent heterogeneity in mind from the outset (Liu et al., 2016; Payne, 2015). These approaches are complementary across both genotype breadth and cellular stage: focused isogenic comparison in neural stem cells resolves NGLY1-attributable effects at the progenitor stage with high confidence (Shyr et al., 2025), whereas the multi-genotype comparison of differentiated neurons presented here maps between-background heterogeneity at a functional, network-forming stage that progenitor models cannot capture.

As noted above, the central constraint of this comparative design is that genotype and genetic background are confounded, and the prioritized signatures have not yet been evaluated within matched genetic backgrounds. Building directly on that limitation, our immediate next direction is to incorporate isogenic gene-corrected controls to determine which of the features identified here are directly attributable to NGLY1 variation rather than to background-dependent variation. Rescue-based designs in single backgrounds have established the feasibility and value of this approach in NGLY1 neurons (Manole et al., 2023). Our own prior work applied CRISPR-corrected isogenic controls to R401X iPSC-derived neuronal stem cells to isolate NGLY1-attributable stress phenotypes (Shyr et al., 2025), establishing this correction strategy for one of the genotypes studied here. Extending it to post-mitotic glutamatergic neurons, and across all three genotypes, with matched transcriptomic, proteomic, and functional profiling remains a substantial and distinct undertaking, and is the logical next step to convert the candidate routes described here into validated, mutation-attributable neuronal signatures.

A second boundary of the present work, complementary to the genotype-background confound, is the restriction of analyses to a single neuronal lineage. Although glutamatergic neurons capture processes central to the neurological manifestations of NGLY1 deficiency, the extent to which the genotype-resolved signatures generalize across other neuronal subtypes, non-neuronal populations, and more complex developmental contexts remains unknown. Brain organoid models constitute a complementary next direction and would allow these signatures to be evaluated within more physiologically relevant and cellularly diverse environment. Together with isogenic validation, this represents the natural extension of the discovery framework established here.

A final consideration concerns the enzymatic consequences of NGLY1 loss. Although NGLY1 transcript and protein abundance were reduced in the patient-derived neuronal cultures (Figure 3), NGLY1-dependent deglycosylation activity was not directly assessed. Residual enzymatic activity may vary across NGLY1 genotypes (He et al., 2015), and reduced transcript or protein levels should therefore not be interpreted as evidence of complete loss of enzymatic function. Direct assessment of genotype-resolved catalytic activity in differentiated neurons is an important component in future validation.

## Conclusion

In conclusion, by comparing patient-derived glutamatergic neurons from three distinct NGLY1 genotypes head-to-head across transcriptomics, proteomic, and functional layers, we show that NGLY1 deficiency does not produce a uniform neuronal phenotype. Instead, distinct trajectories that share only a limited common core yet converge on impaired network maturation. These findings establish a framework for mechanistic subgrouping and for the rational selection of patient-derived models and validation targets in NGLY1 deficiency. Because genotype and background are confounded in this comparative design, isogenic gene-corrected controls are the essential next step to determine which of the prioritized features are directly attributable to NGLY1 variation. More broadly, this works underscores the importance of accounting for genetic heterogeneity when investigating disease mechanisms in rare neurodevelopmental disorders, where overlapping clinical phenotypes may mask substantially divergent underlying biology.

## Data Availability

Transcriptomics data is available in GEO database under the accession number: GSE330278. The proteomics data is available in https://doi.org/10.5281/zenodo.22817557.

## Author disclaimer

The contributions of NIH authors were made as part of their official duties as federal employees and are considered Works of the United States Government. The content of this publication does not necessarily reflect the views or policies of the NIH or the U.S. Department of Health and Human Services.

## Supplementary material

SUPPLEMENTARY FIGURE S1 The RNA-protein discovery gap reflects both attenuated protein-level effects and limited proteomic coverage of low-abundance transcripts. Analyses use the matched molecular universe of gene products detected by both assays across the three disease lines (519D, 592C, 594D). **(A)** Threshold sensitivity of the RNA-protein discovery gap, showing the percentage of significant features called by each assay across a range of FDR thresholds (FDR < 0.01, 0.05, 0.1) and absolute effect-size requirements (no fold-change cutoff, >= 1.25-fold, >= 1.50-fold, >= 2.00-fold). RNA-seq recovered a greater proportion of significant features than proteomics across essentially all threshold combinations and lines. **(B)** Fate of significant RNA changes (RNA DEGs, FDR < 0.05 and |log_2_FC| >= 1) as they pass through the proteomics workflow, expressed as a percentage of RNA DEGs per line. Categories capture the outcome of each cognate protein, including concordant significant, discordant significant, several classes of failed or incomplete protein measurement (failed identification QC, incomplete quantification, insufficient peptide evidence, no human protein identified), and RNA-only outcomes with either an attenuated or an equivalent or stable protein response (protein equivalence margin +/- 0.585 log_2_FC). The large majority of RNA DEGs had no confidently identified cognate protein. **(C)** RNA-only changes and their corresponding protein estimates for representative gene products in each line. Triangles denote RNA effect sizes and circles with horizontal lines denote protein point estimates and 95% confidence intervals; purple shading marks the +/- 0.585 log_2_FC equivalence region. Many transcripts with large RNA fold changes had protein estimates whose confidence intervals fell within or near the equivalence region. **(D)** Expected protein discoveries under a simulation in which protein effect sizes equalled the observed RNA effects, using 500 simulations that preserve per-protein standard errors, shown separately for all jointly tested proteins and for high-confidence proteins. Diamonds indicate the observed number of differentially abundant proteins (DAPs). Observed DAP counts fell well below the counts expected if protein effects matched RNA effects, indicating genuine attenuation rather than power alone. This simulation preserves per-protein precision but not correlation among proteins. **(E)** Proteomic coverage as a function of transcript abundance, showing the fraction of RNA genes whose cognate protein passed QC and entered the limma model across RNA mean-expression deciles (1 = lowest, 10 = highest). Coverage increased monotonically with expression, so low-abundance transcripts were substantially under-represented in the proteomic data.

SUPPLEMENTARY FIGURE S2 Global differential exon-usage burden and targeted analysis of NGLY1, UPR, ER-proteostasis and NMD pathways. **(A)** Number of genes with significant gene-level DEU and number of significant exon bins (bin-level FDR) per disease line. 592C showed the greatest burden, followed by 594D and 519D. **(B)** Shared and line-specific DEU genes across the three lines, based on gene-level FDR with ambiguous multi-gene groups excluded. A large fraction of DEU genes was shared between 592C and 594D or across all three lines, with substantial line-specific sets in 592C and 594D. **(C)** Targeted heatmap of exon-usage changes for selected regulators grouped as NGLY1, XBP1 and ERN1, UPR and ER-proteostasis effectors, and core NMD machinery. Cell shading encodes the gene-level DEU q-value as -log_10_, numbers indicate significant exon bins, asterisks denote gene-level FDR < 0.05, and grey indicates genes not retained or tested. Exon-usage changes in these pathways were most extensive in 592C.

SUPPLEMENTARY FIGURE S3 Cross-omics pathway concordance in 519D. Enrichment results for the all, up, and down marker sets tested independently across RNA-seq, proteomics, and the concordant marker set, organized by pathway database (GO Biological Process, GO Molecular Function, GO Cellular Component, and Reactome). Circle size encodes the number of overlapping genes and colour encodes -log_10_ FDR. Pathways are prioritized by cross-layer coverage and then by FDR, and blank cells indicate no FDR-significant enrichment in that layer. FDR is corrected within each marker set and pathway database.

SUPPLEMENTARY FIGURE S4 Cross-omics pathway concordance in 592C. Enrichment results plotted as in Figure S3, showing marked upregulation of translation, ribosome and rRNA processing terms alongside downregulation of synaptic and neuronal terms.

SUPPLEMENTARY FIGURE S5 Cross-omics pathway concordance in 594D. Enrichment results plotted as in Figure S3, showing predominant downregulation of trans-synaptic signalling, receptor and neuronal system pathways together with extracellular matrix and developmental lineage programs.

SUPPLEMENTARY FIGURE S6 Early multi-electrode array raster plots of network activity across NGLY1 disease lines and control. Representative raster plots of spontaneous activity recorded on multi-electrode arrays for control and the three disease lines (594D, 519D, 592C) at early time points (DIV11, DIV15, DIV20, DIV25, DIV29). Each panel shows spikes across electrodes over a 600 s recording window, with rows corresponding to individual electrodes. Columns denote cell line and rows denote developmental stage in days in vitro (DIV).

SUPPLEMENTARY FIGURE S7 Late multi-electrode array raster plots of network activity across NGLY1 disease lines. Representative raster plots of spontaneous multi-electrode array activity for the three disease lines (594D, 519D, 592C) at later time points (DIV32, DIV34, DIV36, DIV41, DIV43), plotted as in Figure S6 over a 600 s recording window. Columns denote cell line and rows denote developmental stage in DIV.

SUPPLEMENTARY FIGURE S8 Quantitative multi-electrode array metrics reveal line-specific network maturation deficits. **(a)** Developmental trajectories of general activity metrics across DIV for control and the three disease lines (control, blue; 519D, red; 592C, orange; 594D, green), showing number of active electrodes, weighted mean firing rate (wMFR, Hz), and number of bursts. Open circles are individual recordings and solid lines are group trends. **(b)** Developmental trajectories of network-level metrics across DIV, showing number of network bursts and spikes per network burst, plotted as in (a). 592C displayed a distinctive pattern of few network bursts but very high spikes per network burst, whereas 594D and 519D showed more frequent network bursting. **(c)** Representative 600 s raster plots with the population activity trace above for 594D, 519D, and 592C, illustrating characteristic network burst structure per line (blue marks denote detected bursts). **(d)** Expanded raster views over shorter windows (594D and 519D, 200 to 300 s; 592C, 0 to 100 s) resolving individual network bursts, with detected bursts and network bursts highlighted.

SUPPLEMENTARY FIGURE S9 Differential expression of ionotropic glutamate and GABA receptor subunits and related channels across NGLY1 disease lines. Heatmap-style dot plots of log_2_ fold change for ionotropic glutamate receptor (iGluR) subunits, GABA receptor (GABA) subunits, and related ligand-gated channel genes for each disease line (594D, 519D, 592C) at the transcriptomic level (RNA-seq, left three panels, x-axis range approximately -5 to 5) and the proteomic level (proteomics, right three panels, x-axis range approximately -1.5 to 1.0). Point colour encodes the direction of change (up or down) and significant features (DEG status) are indicated. The compressed proteomic fold-change range relative to RNA-seq is consistent with the genome-wide attenuation of protein-level effects.
